# Rheology and Gelation of Hyaluronic Acid/Chitosan Coacervates

**DOI:** 10.3390/biom12121817

**Published:** 2022-12-05

**Authors:** A. Basak Kayitmazer, Fatih Comert, Henning H. Winter, Phillip B. Messersmith

**Affiliations:** 1 Department of Chemistry, Bogazici University, Istanbul 34342, Turkey; 2 Department of Chemical Engineering, University of Massachusetts Amherst, Amherst, MA 01003, USA; 3 Department of Polymer Science & Engineering, University of Massachusetts Amherst, Amherst, MA 01003, USA; 4 Department of Bioengineering, University of California, Berkeley, CA 94720, USA; 5 Department of Materials Science and Engineering, University of California, Berkeley, CA 94720, USA; 6 Materials Sciences Division, Lawrence Berkeley National Laboratory, Berkeley, CA 94720, USA

**Keywords:** coacervation, linear viscoelasticity, rheology, catechol, DOPA, hyaluronic acid, chitosan

## Abstract

Hyaluronic acid (HA) and chitosan (CHI) are biopolyelectrolytes which are interesting for both the medical and polymer physics communities due to their biocompatibility and semi-flexibility, respectively. In this work, we demonstrate by rheology experiments that the linear viscoelasticity of HA/CHI coacervates depends strongly on the molecular weight of the polymers. Moduli for coacervates were found significantly higher than those of individual HA and CHI physical gels. A remarkable 1.5-fold increase in moduli was noted when catechol-conjugated HA and CHI were used instead. This was attributed to the conversion of coacervates to chemical gels by oxidation of 3,4-dihydroxyphenylalanine (DOPA) groups in HA and CHI to di-DOPA crosslinks. These rheological results put HA/CHI coacervates in the category of strong candidates as injectable tissue scaffolds or medical adhesives.

## 1. Introduction

Complex coacervates are a class of materials formed by liquid–liquid phase separation between oppositely charged macroions. The macroions range from organic macromolecules such as proteins [1,2], surfactant micelles [3,4], polypeptides [5], synthetic [6] and biological [7,8] polyelectrolytes to inorganic nanoparticles [9]. The driving forces for complex coacervation are electrostatic interactions and entropic gain due to the release of counterions [5,10]. Recent theoretical and simulation studies have shown that, in addition to the direct Coulombic interactions, charge regulation and charge patchiness are two important contributors to the free energy of interaction. Fluctuations in charges lead to charge regulation [11,12,13], which results in charge inversion on macromolecules such as proteins. On the other hand, charge patchiness arises from the inhomogeneity of charge distribution of a macromolecule [14,15,16]. These two factors make attraction between like-charged macroions possible when a polyelectrolyte is bound to a protein on the *wrong* side of the isoelectric point. Meanwhile, the versatility of coacervation as a macro- or nano-encapsulation platform makes coacervates very attractive for industrial applications such as masking of flavors and oil [17], immobilization of enzymes [18], and controlled delivery of drugs [19], hormones [20] and angiogenic growth factors [21].

Rheology has recently been recognized as a powerful tool that provides information on chain and/or network dynamics within coacervates [6,22]. Experiments on poly(diallyldimethyl ammonium chloride) (PDADMAC)/bovine serum albumin (BSA) coacervates [23] indicated a correlation between relaxation time and parameters affecting the strength of coacervation such as the molecular weight of chains, pH and the ionic strength of the medium. Further rheological studies have included complex coacervation between flexible synthetic polyelectrolytes. Chain dynamics was modeled by “sticky” Rouse or Zimm models, for which chain dynamics was controlled by relaxation of “sticky points” due to transient ion–ion pairs on the chains [22].

The aim of this study is to determine the rheological behavior of complex coacervates formed between oppositely charged biopolyelectrolytes of anionic hyaluronic acid (HA) and cationic chitosan (CHI). HA/CHI coacervates are of major interest for several reasons: (1) The main requirements for medical adhesives are biocompatibility, a high adhesion strength to wet tissue, and the capability to solidify in the time scales required for a surgical operation [24]. Tissue scaffolds should also be biocompatible and biodegradable with non-toxic byproducts, and have a porous structure [25,26,27]. The HA/CHI coacervate constitutes an ideal candidate as a biomaterial since both polymers are biocompatible and non-toxic, suggesting possible applications as scaffolds or wet tissue adhesives. For example, we have recently shown [28] that cells encapsulated by HA/CHI coacervate-based scaffolds are highly viable (greater than 84%). The chondrogenic induction of HA/CHI coacervate-encapsulated cells by TGF-β1 growth factor leads to a remarkable increase in chondrogenic markers [29]. (2) Most of the studies focusing on the rheological properties of coacervates are based on chains that are flexible, as in vinyl-based synthetic polyelectrolytes, or in other cases, where at least one of the polymers is flexible. In contrast, HA and CHI are both semi-flexible, i.e., their intrinsic persistence lengths are 4 nm and 6.5 nm, respectively [30,31]. We have previously shown that coacervation is enhanced for semi-flexible chains [8]. This phenomenon was attributed to a smaller conformational entropy loss for semi-flexible chains, compared to flexible chains, when the chains go from a free solution (unbound) state to a more restricted (bound) conformation, i.e., the degree of freedom of a semi-flexible polyelectrolyte is much less than that of a flexible polyelectrolyte. The semi-flexibility of HA and CHI chains is also one of the reasons why coacervation for a HA/CHI pair takes place at nonstoichiometric charge ratios, in contrast to the stoichiometry observed between flexible polymers [32]. The other reason is that HA has a higher charge spacing than CHI, i.e., 1.3 nm and 0.6 nm, respectively. The inequivalence in charge spacing is assumed to result in loops, where water molecules accumulate and prevent the formation of “contact ion-pairs”, which favor coacervation even at nonstoichiometric charge ratios. The presence of other charge-asymmetric coacervates has also been shown by computational [33] and experimental studies of the poly(ethyleneimine)/polypeptide system [34] and between polyacrylamides with ammonium and sulfate groups [35]. In conclusion, our study here, which is a continuation of our past work [8], provides a deeper insight into the rheological behavior of non-stoichiometric complex coacervates at near physiological pH and salt concentration compared to the high salt concentrations in the literature [36].

In the last part of this work, it is shown how viscoelastic HA/CHI coacervates can be hardened by establishing covalent linkages between catechol-conjugated CHI and HA. Catechols such as L-DOPA are known to stick to both dry and wet surfaces [37]. In fact, marine mussel proteins contain up to 20–30% L-DOPA in their sequence, which is responsible for their strong adhesion in harsh environments [38]. Here, it should be emphasized that polymers conjugated with catechol groups have been found to be highly biocompatible. For example, hydrogels prepared from catechol-modified glycol chitosan showed hemostatic properties with a low immune response and cytotoxity [39]. Meanwhile, cells such as human primary hepatocytes [40] and human adipose-derived stem cells [41] encapsulated with catechol–HA hydrogels exhibited an improved viability compared to those encapsulated in conventional HA hydrogels. Another study [42] has demonstrated a greater than 90% cell viability for Humboldt squid beak protein-mimicking peptide-based coacervates crosslinked with 4-methyl catechol and an oxidant.

In summary, our paper includes a mechanical evaluation of HA/CHI coacervates in the presence and absence of catechol groups on polymer chains. We examine the effect of molecular weight on the rheological properties of HA/CHI coacervates. Regarding HA–catechol/CHI–catechol coacervates, we use a strong oxidant, namely, periodate, to turn coacervates into hardened gels due to the formation of polyphenol crosslinks between HA and CHI chains containing oxidized catechol units, i.e., o-quinone groups [43]. In comparison to another recent study [44], where catechol-conjugated HA and CHI form wet films by doctor blade coating and are then examined by tensile tests, in our study, we aim to examine the phase-separated HA/CHI coacervate itself by linear viscoelasticity measurements.

## 2. Materials and Methods

### 2.1. Materials

Ultrapure research-grade sodium hyaluronate (HA) was obtained from LifeCore Biomedical (Chaska, MN, USA). Chitosan (CHI) samples with acetyl percentages (100 × F_A_) of 24%, 42%, 46%, and 63% were generously donated by Dr. Sabina Strand (Norwegian University of Science and Technology). Molecular weights of HA and CHI samples are given in Table 1. NaOH and HCl were obtained from Fisher Scientific. All solutions were made using Milli-q-grade water.

### 2.2. Preparation of Coacervates

1 mg/mL sodium hyaluronate (HA) was added into 1 mg/mL chitosan (CHI) of equal volume to obtain a final pH of 6.70 ± 0.05 and *I* = 150 mM. All samples were dissolved in 150 mM NaCl except CHI with F_A_ = 0.46, which was dissolved in a mixture of 0.1 M acetic acid and 0.05 M NaCl to obtain a final ionic strength of 0.15 M. The turbid solution was centrifuged at 7000 rpm for 20 min to separate the coacervate phase. The supernatant phase was decanted.

### 2.3. Rheology of HA/CHI Coacervates

Small-amplitude oscillatory shear (SAOS) measurements were performed in a Paar Physica MCR 300 (Anton Paar GmbH) rheometer with Peltier temperature control (Peltier plate and a water circulator) and with humidity control using a solvent trap in combination with moist Kimwipes placed around the rheometer platform. A stainless-steel parallel plate with a 25 mm diameter or cone and plate with a 25 mm diameter and 2° cone angle were the fixtures for the experiments. Strain sweep experiments at 10 Hz (62.8 rad/s) or 10 rad/s for a gap width of 0.4–1 mm, depending on the sample, provided the limits for the linear viscoelastic region. Based on these data (Figure S13 in the Supplementary Materials), SAOS frequency sweep experiments were performed at a strain amplitude of 10% for HA/CHI (F_A_ = 0.24) and 1% for HA/CHI (F_A_ = 0.46 or 0.63) coacervates. Shear rate sweep experiments, starting at a lower rate and ending at a higher rate, determined the dependence of the steady-state viscosity on the shear rate. For time sweep experiments of coacervates, from catechol-modified hyaluronic acid and chitosan, a 2% strain and 10 Hz were chosen based on experiences with prior runs of strain sweep and frequency sweeps for this sample. Most of the rheology experiments were performed on the day of preparation of coacervates to minimize any effect of aging. All experiments were done at 25 °C. IRIS Rheo-Hub software (Iris Development LLC, Amherst, MA, USA) was used for the analysis of rheology data [45].

### 2.4. Modification of HA and CHI with Catechol Groups

The synthesis of catechol derivatives and their conjugation to HA and CHI are described thoroughly in the Supplementary Materials section.

### 2.5. Estimation of the Degree of Modification for HA–Catechol and CHI–Catechol

The degree of catechol modification was determined by UV–Vis spectrophotometry (Shimadzu UV-1700) using 3,4-dihydroxycinnamic acid (DOHA) as a calibration standard. DOHA (52.6 mg) was diluted to 25 mL with 0.015 M HCl solution and served as a stock solution. Then, 10×, 40×, 60×, 80×, 100×, and 150× dilutions of stock solution were prepared. Absorbances at 280 nm and 320 nm (catechol and quinone absorb light at these wavelengths, respectively) were measured, and a calibration curve was drawn.

## 3. Results

The experiments were performed at conditions close to physiological ones, i.e., pH = 6.7 and *I* = 150 mM. The choice of pH = 6.7 came about for two reasons: (i) pH = 6.7 is the upper limit for the water or acetic acid solubility of CHI with an F_A_ of 0.24, 0.42, 0.46, and 0.63. (ii) At a higher pH (pH = 7.4, for instance), the catechol group of dopamine in HA, as used in subsequent in situ hardening experiments, would be oxidized to quinones before any coacervation could take place. It should be emphasized here that the coacervate samples were obtained after the centrifugation of the HA/CHI mixture and the decanting of the supernatant phase. The coacervate phase appeared as slightly translucent and cohesive.

In the experiments reported below, HA/CHI coacervates were all prepared in non-stoichiometric charge ratios ([−]/[+]), i.e., charge ratio ≠ 1. Here, the negative groups belong to deprotonated carboxyls of HA, while positive groups belong to protonated amines of chitosan. The charge ratio of [−]/[+] was calculated from Equation (1)

(1) $$\frac{\left[-\right]}{\left[+\right]}=\frac{\frac{m_{\mathrm{HA}}.\alpha_{\mathrm{HA}}}{{\mathrm{MW}}_{\mathrm{HA}}}}{\frac{m_{\mathrm{CHI}}.\beta_{\mathrm{CHI}}.\mathrm{DD}}{{\mathrm{MW}}_{\mathrm{CHI}}}}$$

where $m_{\mathrm{HA}}$ is the mass of HA, $m_{\mathrm{CHI}}$ is the mass of CHI, α is the degree of ionization of HA (Equation (2)), β is the degree of ionization for CHI (Equation (3)), MW_HA_ is the repeat unit molecular weight for HA, MW_CHI_ is the repeat unit molecular weight for CHI, and DD is the degree of deacetylation of CHI (1 − F_A_). The values of the degree of ionization at pH = 6.7 for CHI with different DD are extrapolated from the potentiometric titration data of Sorlier et al. [46]. Meanwhile, HA had a degree of ionization of 1.0 at pH = 6.7 according to our previous potentiometric titration results [8]. The charge ratio values (Table 2) varied between 1.89 and 2.25 depending on the F_A_ of CHI and indicated the non-stoichiometry of the HA/CHI coacervates.

(2) $$\alpha =\frac{\left[{\mathrm{COO}}^{-}\right]}{\left[{\mathrm{COO}}^{-}\right]+\left[\mathrm{COOH}\right]}$$

and

(3) $$\beta =\frac{\left[{\mathrm{NH}}_{3}^{+}\right]}{\left[{\mathrm{NH}}_{3}^{+}\right]+\left[{\mathrm{NH}}_{2}\right]}$$

### 3.1. Effect of Polymer Molecular Weight

**Figure 1:**
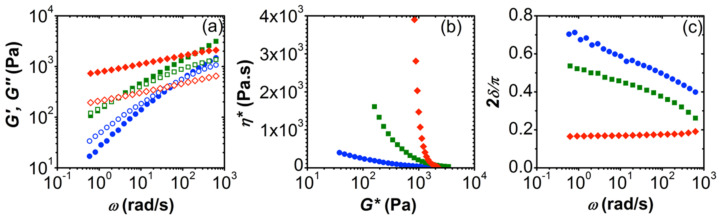
(**a**) Frequency sweep data of HA/CHI coacervates. Closed and open data points are for *G*′ (elastic modulus) and *G*″ (loss modulus), respectively. (**b**) Complex viscosity ($\eta^{*}$) versus complex modulus (G*). (**c**) Normalized phase angle (2δ/π) versus angular frequency (ω ). Red, green, and blue data points represent HA (750 kDa)/CHI (123.5 kDa, F_A_ = 0.42), HA (132.3 kDa)/CHI (365.1 kDa, F_A_ = 0.46), and HA (132.3 kDa)/CHI (123.5 kDa, F_A_ = 0.42) coacervates, respectively.

The material dynamics of HA/CHI coacervates was revealed with small-amplitude oscillatory shear (SAOS) experiments (Figure 1). Compared to physical gels of either HA or CHI at above-entanglement concentrations, HA/CHI coacervates showed more solid-like behavior. For example, HA gels (M_w_ = 1334 kDa) of 10 g/L prepared at pH = 6.05, *I* = 150 mM NaCl had a predominantly viscous behavior (*G*″ > *G*′) up to a high frequency [47] and had a *G*′ value at 10 rad/s around 55-fold lower than that of the HA (750 kDa)/CHI (123.5 kDa) coacervate, i.e., ~20 Pa vs. 1100 Pa, respectively. Meanwhile, gels made solely of chitosan (M_w_ = 850 kDa) prepared at *I* = 0.12 M, pH = 4.2 were also reported to have *G*″ larger than *G*′ at the frequency range studied and had lower values of moduli than those of the HA/CHI coacervates [48]. For instance, at ~10 rad/s, *G*′ is ~10^−1^ Pa and 10^2^ Pa for 10.1 g/L and 41.7 g/ chitosan gels, respectively, while the HA (132.3 kDa)/CHI (332.4 kDa, 63% DD) coacervate had *G*′ = 417 Pa. The observation that *G*″ is predominantly greater than *G*′ in physical gels of HA or CHI alone, while it is the opposite for HA/CHI coacervates (*G*′ is predominantly greater than *G*″), indicates the presence of a stronger network for the latter.

**Figure 2:**
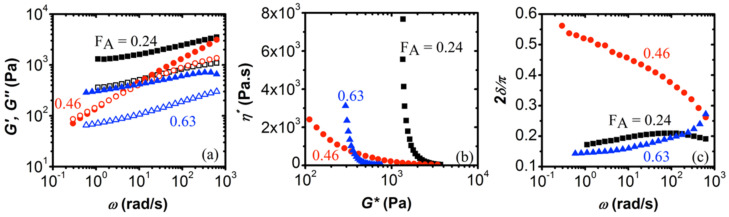
Frequency sweep data for coacervates of HA/CHI with different *F_A_* plotted as (**a**) elastic modulus *G*′ and loss modulus *G*″ vs. frequency ω. The closed and open data points in Figure 2a are for *G*′ and *G*″, respectively. (**b**) Normalized phase angle (2δ/π ) vs. angular frequency (ω). (**c**) Complex viscosity ($\eta^{*}$) vs. complex modulus (G*) for HA/CHI coacervates. Black squares, red circles, and blue triangles are data points for HA/CHI coacervates with F_A_ of 0.24, 0.46, and 0.63, respectively. HA had a molecular weight of 132.3 kDa, while weight-average molecular weight of CHI was similar, i.e., 345.6 kDa, 365.1 kDa, and 332.4 kDa for F_A_ = 0.24, 0.46, and 0.63, respectively.

Polyelectrolyte molecular weight strongly affects the mechanical behavior of HA/CHI coacervates, as seen explicitly in Figure 1. High-molecular-weight HA (M_w_ = 750 kDa) in its coacervates with CHI (M_w_: 123.5 kDa, F_A_ = 0.42) shows a relatively solid-like response within the frequency range studied, while the low M_w_ HA (M_w_ = 132.3 kDa)/CHI (M_w_: 123.5 kDa, F_A_ = 0.42) coacervate shows a viscoelastic one (Figure 1a). Further confirmation of this behavior is seen in complex viscosity ($\eta^{*}$) vs. complex modulus (G*), that is, Winter plot [49,50,51] and normalized phase angle (2δ/π) vs. ω plot in Figure 1b and Figure 1c, respectively. We plot the complex viscosity of small-amplitude oscillatory shear as a function of the complex modulus, the “$\eta^{*}$–G* plot”, to distinguish the phase behavior (solid-like or liquid-like) of our samples (Figure 1b and Figure 2b). The “$\eta^{*}$–G* plot” of the oscillatory shear data serves as a magnifier for structural transitions in complex materials in contrast to the η vs. shear stress (σ) plot in steady shear flow [50]. According to Figure 1b, the solid behavior expresses itself as a distinct upturn at low G*, with a vertical asymptote [50]. The vertical asymptote of $\eta^{*}$ in Figure 1b indicates solid behavior for HA with a M_w_ of 750 kDa. Meanwhile, in the plots of 2δ/π vs. ω, negative slopes at low frequencies represent liquids, while positive slopes at low frequencies represent solids [51]. The solid behavior for the HA chain with the higher molecular weight is confirmed in Figure 1c, with the slightly positive slope of *2*δ/π compared with the negative slope for the liquid.

The molecular weight of chitosan substantially affects the rheological behavior of HA/CHI coacervates. A three-fold increase in the M_w_ of CHI results in a viscoelastic response, where relaxation time (inverse of crossover frequency) increases from 0.07 s to 3.16 s for HA (132.3 kDa)/CHI (123.5 kDa, F_A_ = 0.42) and HA (132.3 kDa)/CHI (365.1 kDa, F_A_ = 0.46) coacervates, respectively. Nevertheless, both samples are in the liquid state, as confirmed by the $\eta^{*}$ vs. G* (Figure 1b) and *2*δ/π vs. ω (Figure 1c) plots. These SAOS experiments indicate that polyelectrolyte molecular weight is an important parameter that directly influences the structure of the HA/CHI coacervates, i.e., longer chains form a highly entangled network where chains relax slowly. Thus, the lifetime of any ionic junctions within the coacervates would be longer for these samples, as expected from entangled polymer solutions.

Relatively more liquid-like behavior for chains of lower molecular weight was also observed for lysozyme/PSS and BSA/PDADMAC coacervates: SANS experiments on the former revealed a fluid structure for short chains and a gel-like structure for long chains [52]. Meanwhile, rheology on BSA/PDADMAC coacervates revealed an increase in terminal relaxation time with M_w_ [23]. The increase in moduli with M_w_, as seen in Figure 1, was attributed to a higher polymer volume fraction within coacervates of longer chains [53]. With regard to polyelectrolyte/polyelectrolyte coacervates, it was reported [22] that the viscoelastic relaxation time of poly(acrylic acid)/poly(N,N-dimethylaminoethyl methacrylate) coacervates increased with polymer chain length, as in our case. Lastly, an increase in relaxation time with polymer molecular weight was reported [54] using PDADMAC/poly(isobutylene-alt-maleate sodium) (IBMA-Na) coacervates. The increase in relaxation time with M_w_ was attributed to the increase in activation energy necessary “for an ionic pair to dissociate from bond length to Debye screening length”. In conclusion, our results, which are in line with the literature on protein/polyelectrolyte and other polyelectrolyte/polyelectrolyte coacervates, indicate stronger interchain connectivities with increasing M_w_, leading to the slowing down of the chains and consequential increase in the relaxation times.

### 3.2. Effect of Degree of Acetylation

The results of the frequency sweeps in the linear viscoelastic regime are shown in Figure 2 for HA (132.3 kDa)/CHI coacervates of varying degrees of acetylation (F_A_). The weight-average molecular weights of CHI were 345.6 kDa, 365.1 kDa, and 332.4 kDa for F_A_ = 0.24, 0.46, and 0.63, respectively.

According to Figure 2a, coacervates of HA with CHI of F_A_ = 0.24 and F_A_ = 0.63 gave a highly elastic response in the frequency range studied, i.e., *G*′ > *G*″. Thus, these coacervates are considered to be elasticity-dominated networks. Similar behavior has been observed for coacervates of BSA/pectin [55] and fish gelatin/sodium montmorillonite [56]. On the other hand, the HA/CHI coacervate with F_A_ = 0.46 has a more dominant *G*″ over *G*′ at low frequencies. Such viscoelastic behavior is also observed in the case of coacervates of poly(D,L-glutamic acid) or poly(D,L-aspartic acid) with polyethyleneimine [34] and coacervates of poly(N,N-dimethylaminoethyl methacrylate) (PDMAEMA) with PAA [53].

The distinction between solid and liquid behavior is more apparent in Figure 2b,c. The abrupt negative slope of the phase angle (2δ/π) vs. ω plots for the HA/CHI coacervate with F_A_ = 0.46 represents liquid behavior. The other samples are soft solids near the gel point, i.e., the loss angle is nearly flat at low frequencies (Figure 2b). We find the lowest G* values of 1370 Pa and 320 Pa for HA/CHI coacervates with F_A_ = 0.24 and 0.63, respectively (Figure 2c). On the other hand, the coacervate with F_A_ = 0.46 is devoid of the low-frequency upturn, indicating its liquid character. At a high G*, the three samples are very similar.

The results above were quite surprising, as our previous work [8] on the coacervation of HA/CHI had revealed a linear trend of $pH_{\varphi }$, i.e., pH for the onset of coacervation, with F_A_. Thus, we would expect that chitosan chains with a higher degree of acetylation, which corresponds to a lower polyelectrolyte charge density, would have weaker electrostatic interactions with HA. This would, then, lead to more liquid-like materials. Namely, chitosan with F_A_ = 0.63 has a lower charge density than the other two samples but a stronger degree of hydrophobicity due to its higher amount of acetyl groups, which would explain why the elastic moduli are higher than the loss moduli in the frequency range studied. In addition, it is likely that the acetyl groups in the CHI sample with F_A_ = 0.63 had a blocky distribution since we had to use acetic acid to dissolve it, while the other CHI samples with F_A_ of 0.24 and 0.46 were easily dissolved in NaCl solution. Even a slightly higher distribution of blocky acetyl groups would enhance the hydrophobic interactions and contribute to the observed solid-like behavior. In the future, we are planning to extend this study to chitosan samples with a wider range of F_A_s.

### 3.3. Shear-Dependent Behavior of HA/CHI Coacervates

**Figure 3:**
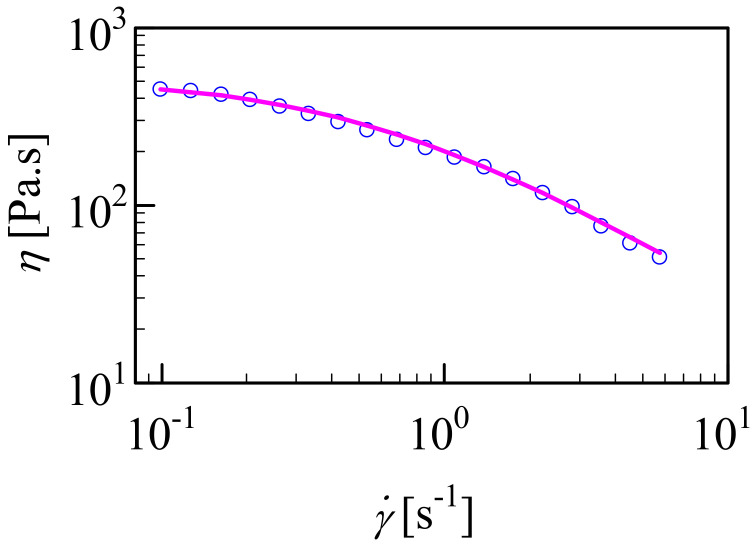
Shear viscosity versus shear rate for HA (M_w_: 132.3 kDa)/CHI (F_A_ = 0.42) coacervate. Line shows fitting of the data to Carreau model given in Equation (4).

It is important to know the shear rate-dependent behavior of HA/CHI coacervates for their applications as biomaterials. We selected HA (132.3 kDa)/CHI (123.5 kDa, F_A_ = 0.42) as its liquid-like behavior made it suitable for shear rate sweep measurements (Figure 3). Compared to the concentrated HA and CHI solutions alone, the viscosity at 1 Hz of the HA/CHI coacervate was much higher, i.e., 211 Pa.s for the HA/CHI coacervate vs. 40 Pa.s for 20 mg/mL HA with M_w_ = 150 kDa [57] and 0.57 Pa.s for 10 mg/mL chitosan of F_A_ = 0.372 and M_w_ of 550 kDa [58]. Thus, the high viscosity of the HA/CHI coacervate cannot be attributed solely to the high concentration of HA and CHI chains within the coacervate.

The shear sweep data were best fitted with the simplified Carreau model [59].

(4) $$\eta \left(\dot{\gamma }\right)=\frac{\eta_{0}}{(1+\tau {\dot{\gamma )}}^{n}}$$

where $\eta_{0}$ (Pa.s) is the limit viscosity at low shear rates, τ is a characteristic time for the fluid, $\dot{\gamma }$ is the shear rate (s^−1^), and *n* is the “Carreau exponent”. The Carreau fit shown in Figure 3 gave $\eta_{0}=$ 526 Pa.s, τ = 1.76 s, and *n* = 0.945, which indicates extremely strong shear thinning.

As observed with many other examples in the literature [22,56,60,61,62,63,64], we observed shear thinning of coacervates as the shear rate was increased. The shear thinning behavior of the HA/CHI coacervates is very encouraging for their future application as biomaterials, e.g., through a syringe needle or catheter with low pressure [60].

The mechanism behind the shear thinning behavior is usually attributed to the alignment of the labile network of coacervates along the shear force. Here, we presume that HA/CHI coacervate networks are formed of clusters where HA and CHI chains are in loosely associated contact pairs. The loose association is assumed to arise from conformational restrictions due to the semi-flexibility of the chains and the charge inequivalence between HA and CHI. At high shear rates, these clusters are elongated.

### 3.4. Conversion of Complex Coacervates into Chemical Gels

**Figure 4:**
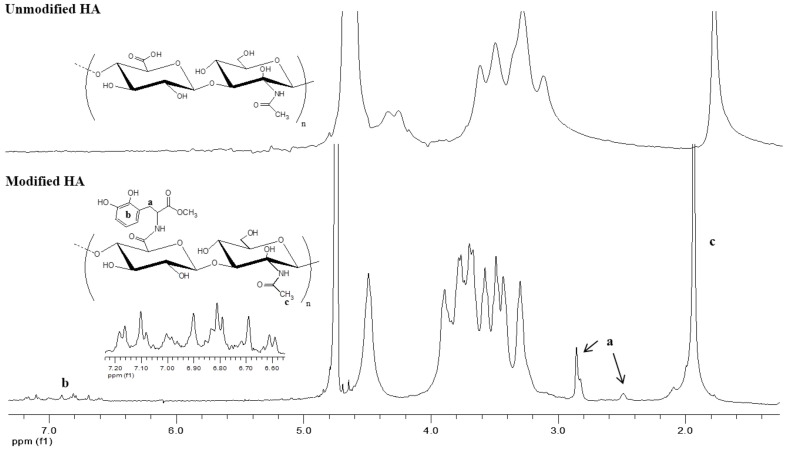
^1^H NMR of hyaluronic acid–catechol (modified HA) versus unmodified hyaluronic acid (HA) in D_2_O.

**Figure 5:**
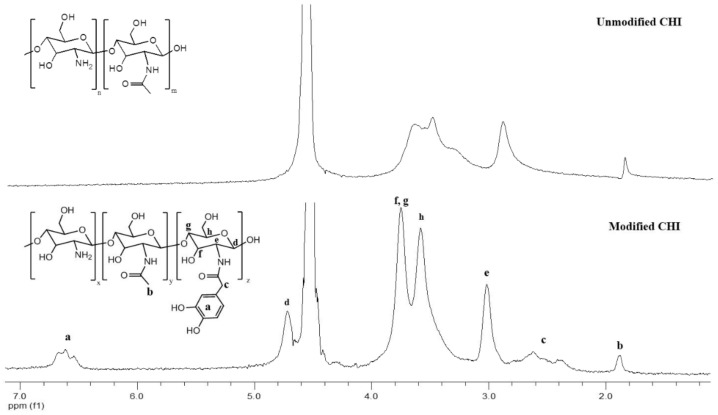
^1^H NMR of chitosan–catechol (modified CHI) versus unmodified chitosan (CHI) in D_2_O.

As much as complex coacervates present stronger materials than physical gels in terms of their rheological response, covalently linked systems usually provide opportunities to obtain materials with even higher strength. For this purpose, we first modified HA and CHI with catechol groups. As described in the Supplementary Materials, the modification reactions were both based on carbodiimide chemistry. In the case of HA, the carboxylic acid groups of the polymer reacted with 1-Ethyl-3-[3-(dimethylamino)propyl]-carbodiimide (EDC) in the presence of the activating agent N-hydroxysuccinimide (NHS), forming an NHS ester, which later crosslinked to primary amines of L-DOPA methyl ester. For the case of CHI, which was the supplier of primary amines, a similar reaction scheme was applied, except (i) N,N′-Dicyclohexylcarbodiimide (DCC) was used instead of EDC and (ii) carboxyl groups were provided from an acetonide derivative of hydrocaffeic acid, DHPA-(Acet)-OH. The products of the intermediate steps were all confirmed by ^1^H NMR and ESI-MS (Supplementary Materials). Catechol-modified polymers were characterized by ^1^H NMR (Figure 4 and Figure 5). The two different peaks at 6.6–7.2 ppm and 2.9 ppm in Figure 4 belong to the phenyl ring hydrogens and -CH_2_- of L-DOPA, respectively, as confirmed by the ^1^H NMR in Figure S12 of the Supplementary Materials. -CH_2_- peaks between 2–3 ppm and the phenyl ring hydrogen peaks around 6.6 ppm in Figure 5 are for DHPA-functionalized chitosan, as confirmed by the ^1^H NMR in Figure S2 of the Supplementary Materials. UV–Vis measurements provided an estimate for the degree of modification as 1.7% (by mole) and 5.6% for CHI and HA, respectively.

**Figure 6:**
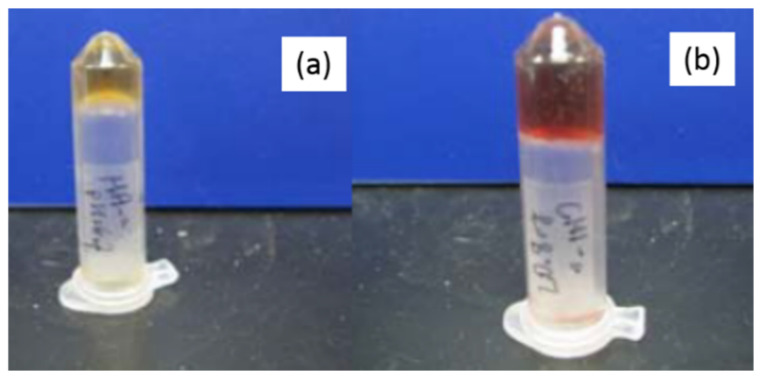
Pictures of polymers gelled by periodate. (**a**) Catechol-modified HA, (**b**) Catechol-modified CHI. The diameter of the tube was approximately 1 cm.

The catechol group of DOPA is known to oxidize in the presence of sodium periodate (NaIO_4_), leading to the formation of di-DOPA, which acts as a crosslinker between polymer chains [65,66]. Modified HA and CHI were dissolved separately in 1× PBS (pH: 7.4) buffer at a concentration of 200 mg/mL, and then sodium periodate (1 mg) was added. The formation of a yellow gel confirmed the presence of catechol groups on the polymer chains. Gelation took less than an hour (Figure 6).

**Figure 7:**
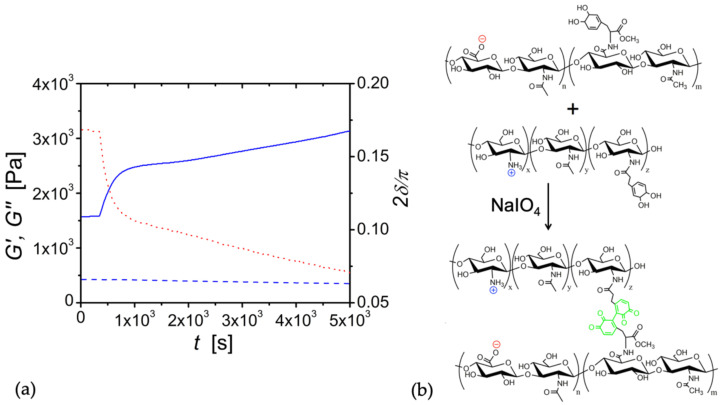
(**a**) Time sweep of CHI (F_A_: 0.42, catechol: 1 mol %)/HA (M_w_: 750 kDa, catechol: 1 mol %) coacervate, to which 1 mM periodate solution was added at the sample rim after 5 min. Frequency = 10 Hz. Strain = 2%. The blue solid line represents *G*′, while the blue dashed line denotes *G*″. The red dotted line represents 2δ/π. (**b**) Schematics of chemical conversion.

With these catechol-modified biopolyelectrolytes, our goal was to establish coacervation followed by gelation. Five minutes after mixing the modified polymers, the addition of periodate solution to the HA/CHI coacervate caused an abrupt increase in the storage modulus, i.e., *G*′ went from 1.59 × 10^3^ Pa.s to 2.45 × 10^3^ after 15 min (Figure 7). The periodate-triggered hardening of the coacervate is expected since periodate creates crosslinks between catechol groups. Thus, coacervation enhanced the chemical crosslinking of HA and CHI by bringing the catechol groups in close proximity to each other. Future studies will involve the tuning of the modulus with different amounts of periodate and varying percentages of catechol grafting of HA and CHI chains.

The results above indicate that chemically crosslinked HA/CHI coacervates are appropriate for use as a biomaterial in several soft tissues. For instance, the grey and white matter of the brain tissue has an elastic modulus (*G*′) of 3.1 kPa and 2.7 kPa, respectively [67], while the sinus wall of pulmonary valves [68] has a *G*′ of 2.85 kPa. As seen in Figure 7, upon the addition of 1 mM NaIO_4_, catechol-modified HA and catechol-modified CHI were shown to crosslink and achieve an elastic modulus of 2.75 kPa within 2870 s, and the modulus increased with time. Therefore, it is possible that these tissue-comparable values of G′ can be easily attained within the time scale of a surgery.

We should also consider the rheological response of HA/CHI coacervates at lower pHs than the physiological pH because cells are slightly acidified in inflammatory diseases [69]. For example, when the pH is dropped from 6.7 to 6.0, the degree of protonation for CHI changes from 0.3 to 0.7, while the degree of deprotonation for HA stays constant at 1.0 [8]. Thus, it can be concluded that the electrostatic interaction between CHI and HA would increase, leading to longer crossover relaxation times and/or a more solid-like response in the case of lower pHs.

Lastly, as mentioned before, the rheology experiments were done at 25 °C. However, we should note that at physiological temperature, HA/CHI coacervates might show lower moduli and viscosity due to the break-up of any hydrogen bonds present. This would mean loosening of the coacervate network and could jeopardize its usefulness as a medical adhesive or filler.

## 4. Conclusions

The main parameter for controlling the mechanical properties of HA/CHI coacervates is the molecular weights of HA and CHI chains. An increase in the molecular weight of either HA or CHI results in the slowing down of the network dynamics, evidenced by either a switch from a viscoelastic to a gel-like response or an increase in relaxation time.

We have also shown that moduli values could be increased by the conversion of coacervates to chemical gels. By the modification of HA and CHI with DOPA groups, covalent crosslinks were created between the two biopolymers in the presence of periodate, and a 1.7-fold increase in moduli was observed within 15 min. Such a level of hardening with time was previously shown only for coacervates made of diblock or triblock polymers [70]. Furthermore, compared to other coacervates that harden with time, the presence of unoxidized catechols as a functional group of HA and CHI make this coacervate system an ideal candidate as a medical adhesive. The potential stickiness of catechol-modified HA/CHI coacervates to wet and dry tissues will be explored in future studies.

## Figures and Tables

**Table 1:** Weight-average (M_w_) and number-average (M_n_) molecular weight of chitosan and hyaluronic acid.

Polymer	M_w_ (kDa)	M_n_ (kDa)
Chitosan, F_A_ = 0.24 ^a^	345.6	125.5
Chitosan, F_A_ = 0.42 ^a^	123.5	104.5
Chitosan, F_A_ = 0.46 ^a^	365.1	302.8
Chitosan, F_A_ = 0.63 ^a^	332.4	209.5
Hyaluronic acid ^b^	750	N/A
Hyaluronic acid ^b^	132.3	N/A

^a^ Determined by size exclusion chromatography (SEC) measurements in citrate buffer at pH 3.50. ^b^ Determined by multi-angle laser light scattering (MALLS) measurements.

**Table 2:** Charge ratio, [−]/[+], of carboxyl groups of HA to amine groups of CHI at different degrees of acetylation (F_A_).

F_A_	MW of Chitosan Repeat Unit (g/mole)	Degree of Ionization * (β) at pH = 6.7	[−]/[+] at pH = 6.7
0.24 0.42 0.46 0.63	196.1 194.9 194.7 193.6	0.34 0.44 0.45 0.58	1.89 1.90 2.00 2.25

* Extrapolated from Sorlier et al. [46]. “Adapted with permission from Ref. [46]. Copyright {2001} American Chemical Society”.

## Data Availability

Not applicable.

## Supplementary Materials

The following are available online at https://www.mdpi.com/article/10.3390/biom12121817/s1, Experimental procedures for the synthesis of catechol-derivatives and their conjugation to HA and CHI including Figure S1: Schematics of DHPA(Acet)-OH production, Figure S2: ^1^H NMR of DHPA-OMe in deuterated-DMSO, Figure S3: ^1^H NMR (400 MHz) for DHPA(Acet)-OMe in DMSO-d_6_, Figure S4: ESI-MS for DHPA(Acet)-OMe in methanol, Figure S5: ^1^H NMR of DHPA(Acet)-OH in DMSO-d_6_, Figure S6: ESI-MS of DHPA(Acetonide)OH in methanol, Figure S7: Schematics of DHPA(Acet)-OH to DHPA(Acet)-OSu, Figure S8: ^1^H NMR of DHPA(Acet)-OSu in Chloroform-d_6_, Figure S9: ESI-MS of DHPA(Acet)-OSu in methanol, Figure S10: Reaction scheme for the preparation of DOPA-OMe, Figure S11: Reaction scheme for the modification of hyaluronic acid with DOPA methyl ester, Figure S12: ^1^H NMR of L-DOPA and L-DOPA methyl ester in D_2_O, Figure S13: Strain sweep data. References [71,72] are cited in the Supplementary Materials.
