# Supplementary material for: Rheology and Gelation of Hyaluronic Acid/Chitosan Coacervates
Source: Biomolecules. 2022 Dec 5;12(12):1817. doi: 10.3390/biom12121817 (PMC9775361; doi:10.3390/biom12121817)
Supplement: Supplementary file 1 [file biomolecules-12-01817-s001.zip › biomolecules-1991800-supplementary.pdf]

Supporting Materials

Rheology and Gelation of Hyaluronic Acid/Chitosan Coacervates

A. B. Kayitmazer[1*], F. Comert[1], H. H. Winter[2], P.B. Messersmith[3,4]

[1] Department of Chemistry, Bogazici University, Istanbul 34342, Turkey
[2]Department of Chemical Engineering and Department of Polymer Science & Engineering, University of Massachusetts Amherst, Amherst, MA 01003, USA
[3]Department of Bioengineering and Department of Materials Science and Engineering, University of California, Berkeley, CA 94720, USA
[4]Materials Sciences Division, Lawrence Berkeley National Laboratory, Berkeley, CA 94720, USA

1. Modification of Chitosan

1.1. Materials

Chitosan (degrees of acetylation 42%) was gifted by Dr. Sabina Strand (Norwegian University of Science & Technology, Trondheim, Norway). 3,4-Dihydroxyhydrocinnamic acid (DHPA), Thionyl chloride, *p*-toluenesulfonic acid monohydrate, 2,2-dimethylpropane (DMP), IronIII(Ferric) chloride, lithium hydroxide, N-hydroxysuccinimide (HOSu), N,N'-Dicyclohexylcarbodiimide (DCC), sodium periodate, ninhydrin reagent solution, silica gel (pore size:60A^o, 70-230 mesh size), and dialysis tubing (MWCO: 10,000 Daltons) were purchased from Sigma-Aldrich. Solvents such as anhydrous benzene, isopropanol, dichloromethane (DCM), ethyl acetate (EtOAc), hexane, tetrahydrofuran (THF), acetic acid, ethanol, acetonitrile (ACN), trifluoroacetic acid (TFA) and triisopropylsilyl (TIPS) were also purchased from Sigma-Aldrich, and used without further purification.

1.2. Synthetic Procedures for Modication of Chitosan

Modification of chitosan with a catechol group was accomplished by a scheme which starts with protection of hydroxyls on the benzene ring by converting the molecule into DHPA(Acetonide)-OH (Figure S1). Products were confirmed by ^{1}H NMR (Varian Mercury-VX 400 MHz BB) and ESI-MS analysis (Finnigan, Thermoquest, CA).

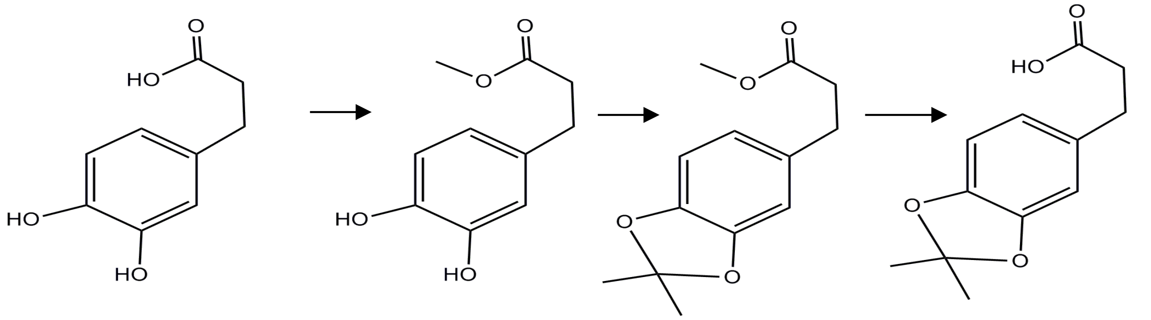

DHPA DHPA-OMe DHPA(Acet)-OMe DHPA(Acet)-OH

Figure S1: Schematics of DHPA(Acet)-OH production.

1.2.1. Synthesis of DHPA-Methyl ester (DHPA-OMe)

This method was adapted from Aubry et al[71]. 18.2 gram (100mmol) DHPA (3,4-Dihydroxyhydrocinnamic acid = 3-(3,4-Dihydroxyphenyl)propionic acid) was dissolved in 250 ml of dry methanol under Argon in an ice/isopropanol bath. Thionyl chloride (14.6 ml, 200mol) was added slowly into the reaction mixture. After stirring for half an hour, the cooling bath was removed and the reaction was allowed to run overnight at room temperature. The mixture was rotavapped to obtain a dark brown oil, which was put under vacuum to yield a dark brown solid (90% yield). Figure S2 provides ^{1}H NMR of DHPA-OMe obtained by this procedure. **DHPA-OMe.** ^{1}H-NMR Spectrum (400 MHz, DMSO-d_6): δ 8.72 (s, 1H), 8.66 (s, 1H), δ 6.55-6.52 (m, 3H), δ 3.55 (s, 3H), δ 2.65 (m, 2H), δ 2.49 (m, 2H).

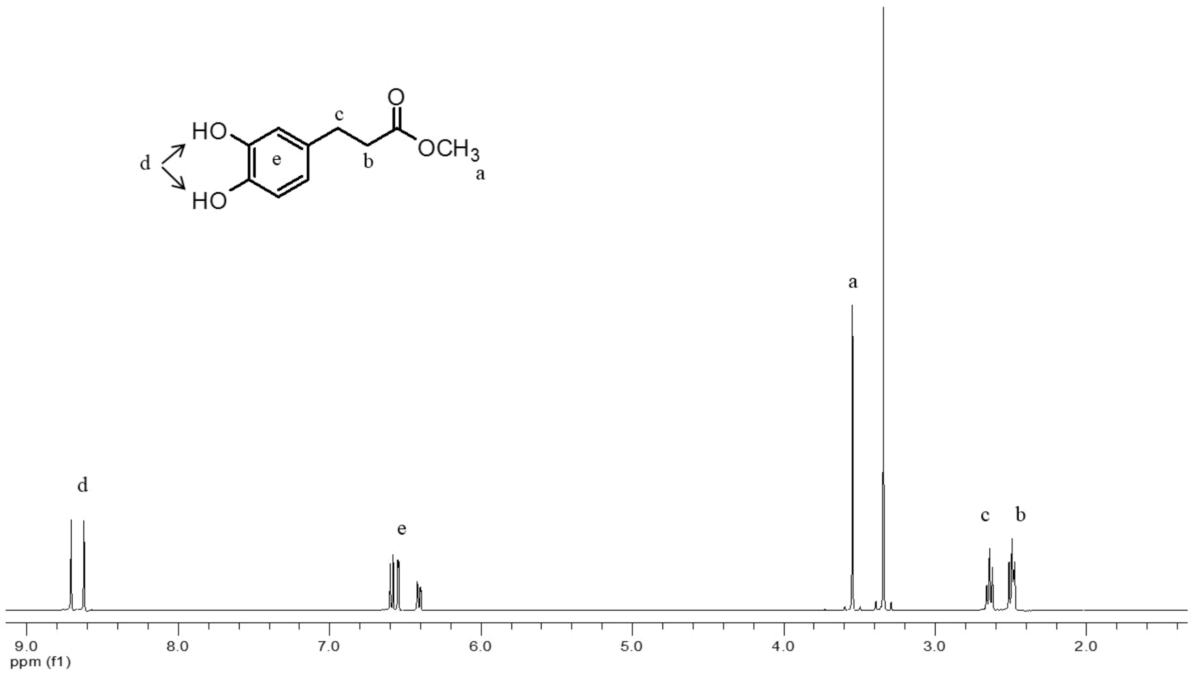

Figure S2: ^{1}H NMR of DHPA-OMe in deuterated-DMSO.

1.2.2. Synthesis of DHPA(Acet)-OMe

This method was adapted from Liu et al[72]. DHPA-OMe (9.8 mg, 50 mmol), DMP (2,2-dimethylpropane) (30 ml, 4 equiv.), and anhydrous benzene (ca. 180 ml) were mixed. Soxhlet extraction was applied where the thimble was loaded with anhydrous $CaCl_2$ to remove the methanol and water formed during the reaction. After a 15-minute degassing with argon, and heating to reflux for another 15 minutes, *p*-toluenesulfonic acid monohydrate (475 mg, 5 mol%) was added as a catalyst. By applying the starting material (DHPA-OMe) and the reaction product on a TLC plate, dipping the plate in 1 % $FeCl_3$ solution in ethanol, and finally heating the TLC plate in an oven, a negative test result was sought. Once confirming the absence of phenol groups by the ferric chloride test, the reaction was ended. The mixture was then rotavapped and purified by preparative column chromatography where it was washed with dichloromethane (DCM)/ethyl acetate (EtOAc). The solution was further purified by silica-gel flash chromatography (washed by DCM/hexane, then eluted with DCM/EtOAc) to yield a colorless oil (20% yield). The purity of

the product was monitored with ^{1}H NMR (Figure S3) and ESI-MS (Figure S4).

DHPA(Acetonide)-OMe. ^{1}H-NMR Spectrum (400 MHz, DMSO-d_6): δ 6.67-6.57 (m, 3H), δ 3.55 (s, 3H), δ 2.71 (m, 2H), δ 2.54 (m, 2H), δ 1.57 (s, 6H).

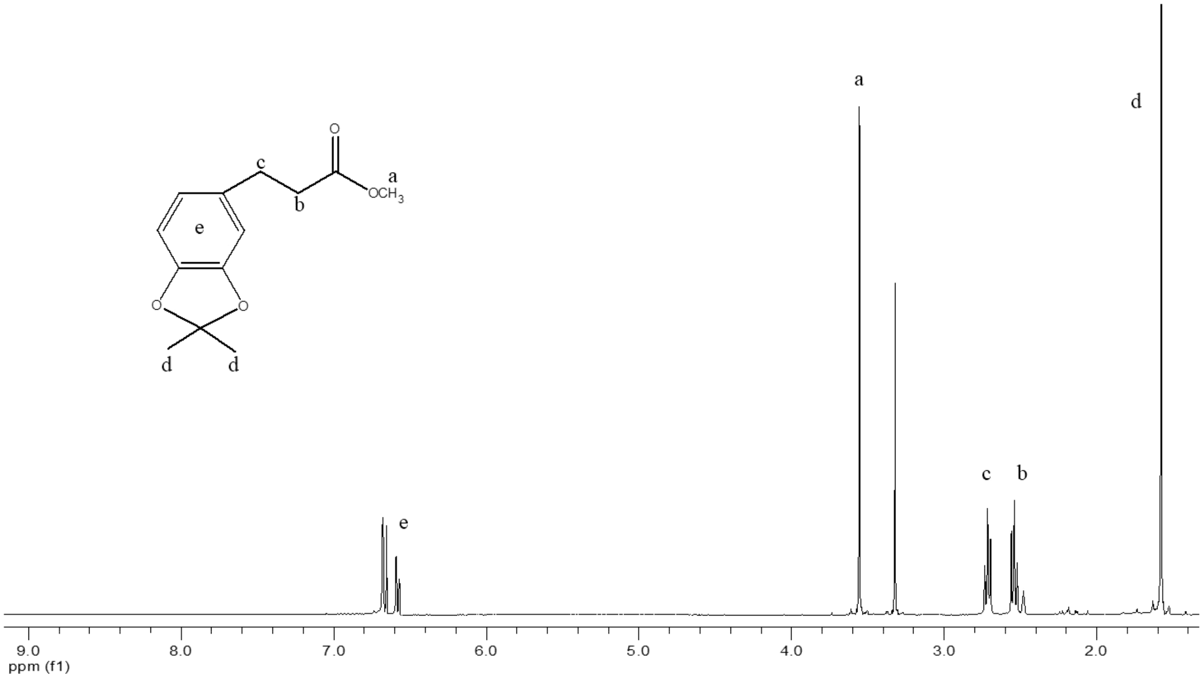

Figure S3: ^{1}H NMR (400 MHz) for DHPA(Acet)-OMe in DMSO-d_6.

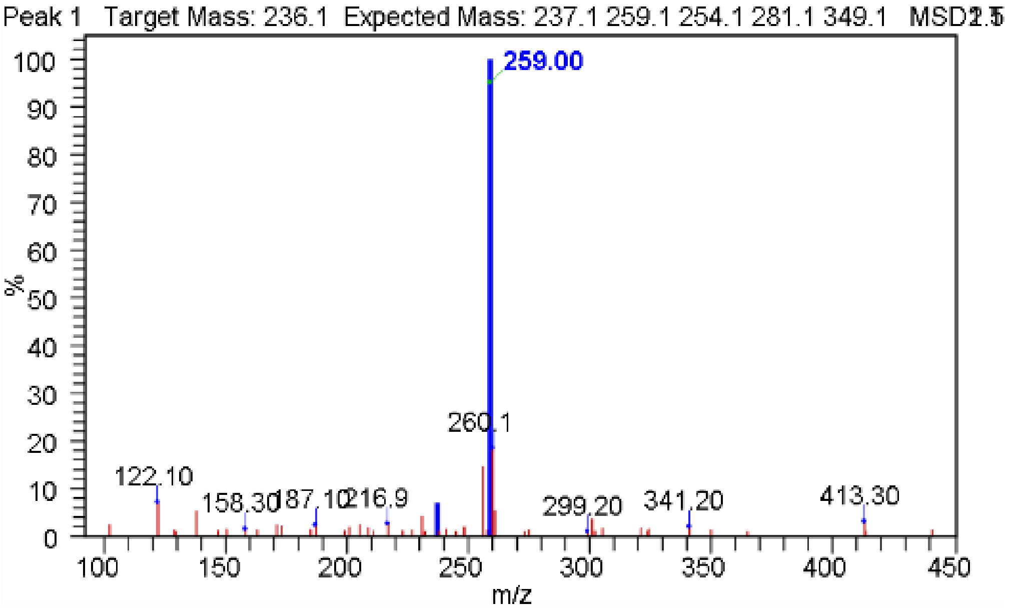

Figure S4: ESI-MS for DHPA(Acet)-OMe in methanol.

1.2.3. Synthesis of DHPA(Acet)-OH

DHPA(Acet)-OMe (5.32g, 1.93 mmol), THF (60 ml), and lithium hydroxide (0.93 g, 2 equiv) solution (20 ml) were mixed together in a 250-ml flask. The mixture was stirred for 6 hours at room temperature. After checking for purity with TLC, the solution was concentrated under reduced pressure, treated with 1 M citric acid to a pH of 5.0, extracted with EtOAc, washed with water, dried over $MgSO_4$, and concentrated again under reduced pressure. The resulting solution was recrystallized in DCM/hexane to afford white crystals, 4.4 g (88% yield). The resulting DHPA(Acet)-OH was characterized by both ^{1}H NMR (Figure S5) and ESI-MS (Figure S6). **DHPA(Acetonide)OH**. ^{1}H-NMR (400 MHz, DMSO-d_6): δ 6.55 (d, 1H), δ 6.52 (s, 1H), δ 6.46 (dd, 1H), δ 2.54 (t, 2H), δ 2.30 (t, 2H), δ 1.44 (s, 6H).

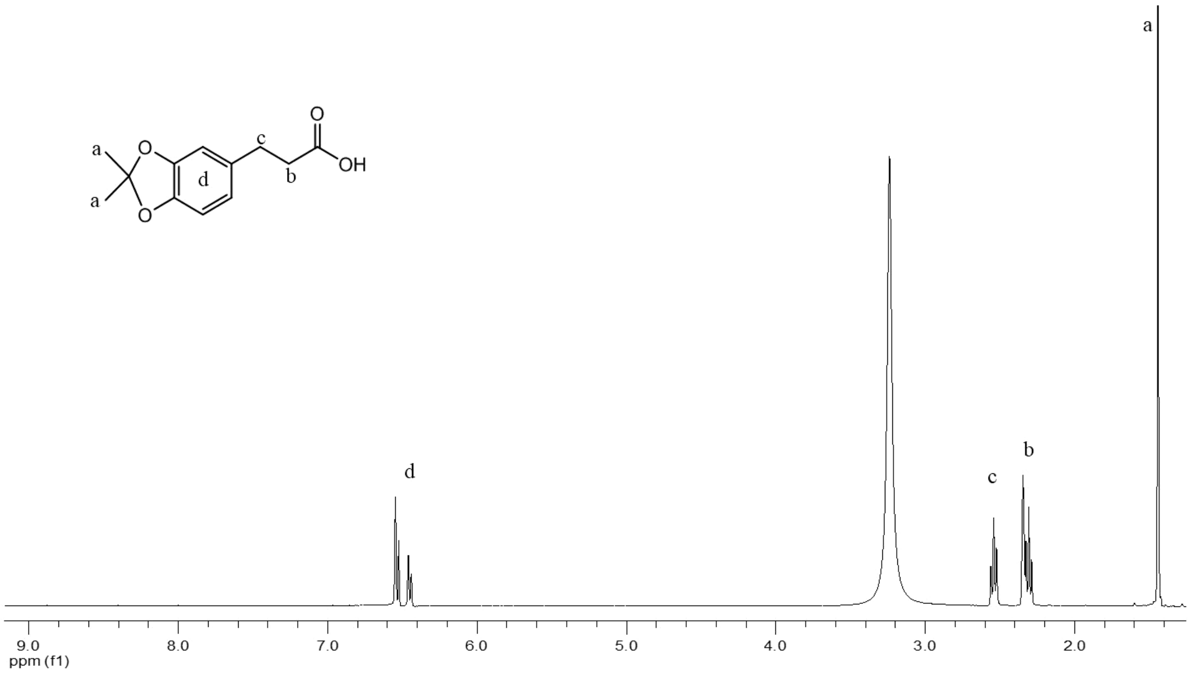

Figure S5: ^{1}H NMR of DHPA(Acet)-OH in DMSO-d_6.

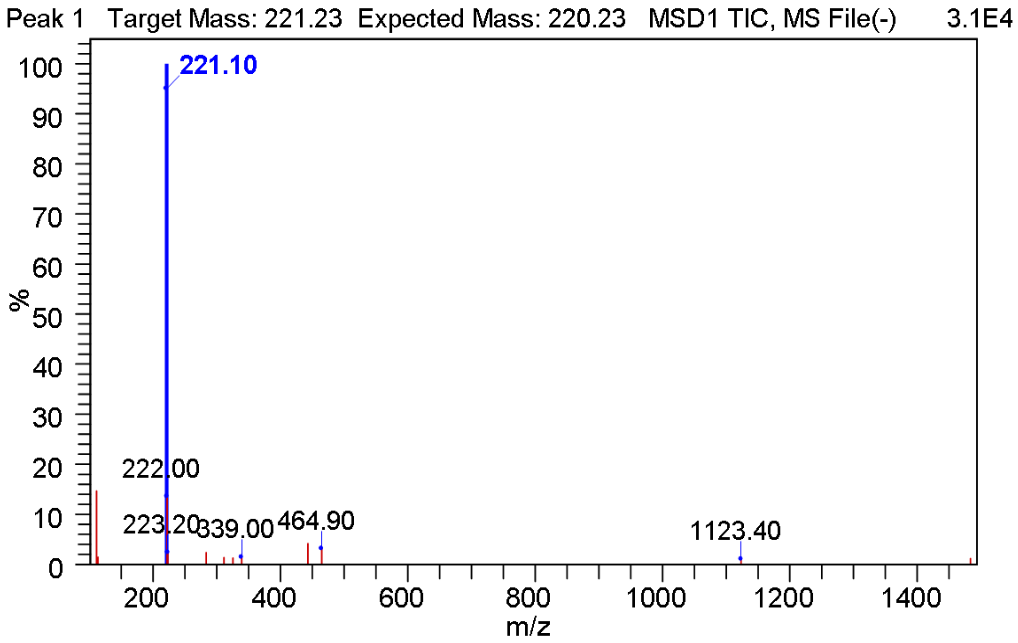

Figure S6: ESI-MS of DHPA(Acetonide)OH in methanol.

1.2.4. Synthesis of DHPA(Acet)-OSu

Acetonide derivative of hydrocaffeic acid (DHPA(Acet)-OH) in DCM was reacted with N-hydroxysuccinimide (HOSu) in acetonitrile (ACN) followed by addition of N,N'-Dicyclohexylcarbodiimide (DCC) dissolved in DCM. The purity of the reaction product was checked with the ferric chloride-treated TLC plates (mobile phase of DCM:MeOH:HOAc 100:6:1). Any unreacted chemicals were removed by re-crystallization. After rotavapping DCM/ACN, the reaction mixture was dissolved in a hot water bath, and isopropanol was added into the mixture gradually. Then the reaction mixture was let to cool down overnight at room temperature while the crystals were forming. Formed crystals were then collected by vacuum filtration and dried by vacuum drying. Figure S7 summarizes the described procedure. Characterization was performed with ^{1}H NMR (Figure S8) and ESI-MS (Figure S9). **DHPA(Acetonide)-OSu.** ^{1}H-NMR Spectrum (400 MHz, DMSO-d_6): δ 6.80-6.70 (m, 3H), δ 2.94 (m, 2H), δ 2.83 (m, 2H), δ 2.81 (s, 2H), δ 1.60 (s, 6H).

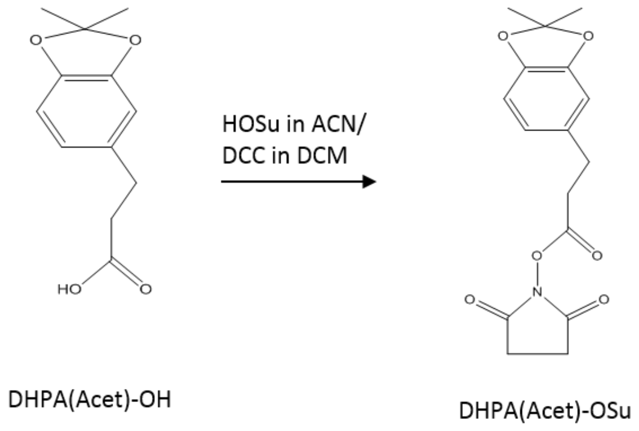

Figure S7: Schematics of DHPA(Acet)-OH to DHPA(Acet)-OSu.

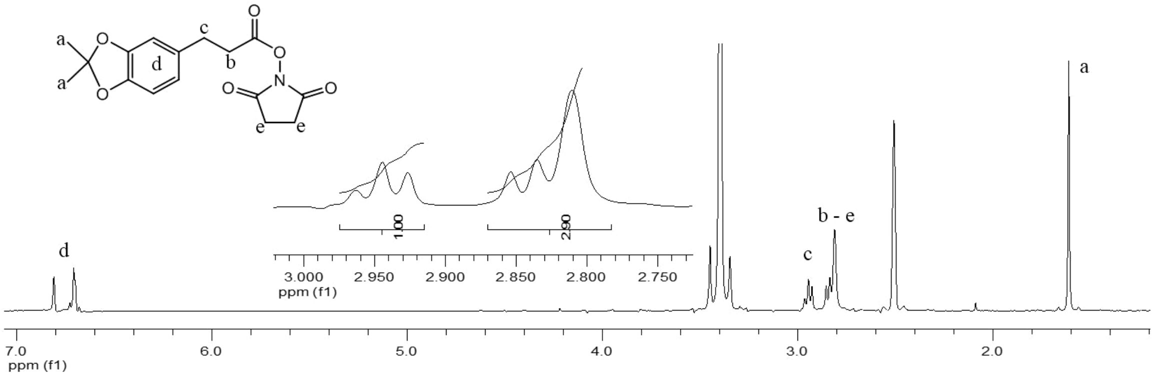

Figure S8: ^{1}H NMR of DHPA(Acet)-OSu in Chloroform-d_6.

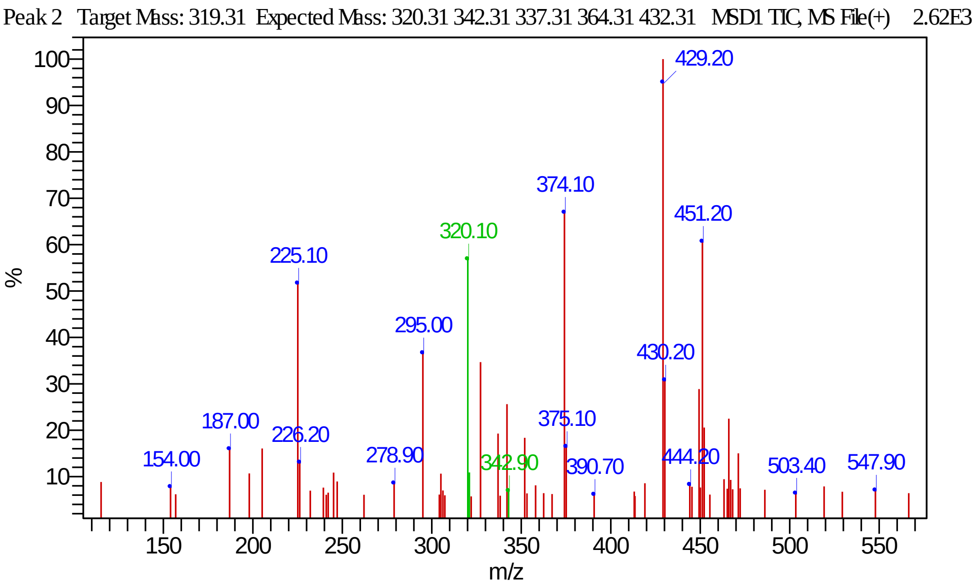

Figure S9: ESI-MS of DHPA(Acet)-OSu in methanol.

1.2.5. Modification of Chitosan with DHPA(Acet)-OSu

250 mg (1.4 mmoles) of chitosan (degrees of acetylation: 42 %) was dissolved in 250 ml of water. Next, the pH of the solution was adjusted to 8.4 by adding 0.1 N NaOH. To this solution, 164.4 mg (1.4 mmoles) DHPA(Acet)-OSu dissolved in 10 ml of ACN was added gradually over 2 hours. After letting the reaction run for 5.5 hours at room temperature, TLC was carried out with DCM: Methanol: Acetic Acid (100:6:1). Following the centrifugation of the reaction, the supernatant was frozen and lyophilized for two days. The cleavage of the acetonide group was accomplished by dissolving the modified chitosan in a solution of trifluoroacetic acid (TFA):triisopropylsilyl (TIPS):H_2O (95:2.5:2.5) for 30 minutes. The mixture was rotavapped before being precipitated with ether. After vacuum-drying for 2 hours, the sample was dissolved in Milli-Q-water at pH = 3.0, and then lyophilized. **Chitosan-Catechol.** ^{1}H-NMR Spectrum (400 MHz, D_2O): δ 6.67-6.54 (m, 3H), δ 4.73 (H_d), δ 3.75 (H_f, H_g), δ 3.58 (H_h), δ 3.02 (H_e), δ 2.62 (br, 2H), δ 2.41 (br, 2H), δ 1.87 (H_i) [br: broad].

2. Modification of Hyaluronic Acid

2.1. Materials

Sodium hyaluronate (M_W: 132.3 kDa) was obtained from Lifecore Biomedical, LLC (Chaska, MN, USA) and used without further purification. N-hydroxysuccinamide (NHS), 3,4-dihydroxy-L-phenylalanine (L-DOPA), 3-(N-Morpholino)propanesulfonic acid (MOPS) and its sodium salt, and toluene were purchased from Sigma Chemical Co. (St. Louis, MO, USA). 1-Ethyl-3-[3-(dimethylamino)propyl]-carbodiimide (EDC) was purchased from Fluka. Methanol (dried max. 0.003% H_2O), thionyl chloride ($SOCl_2$) and sodium metaperiodate ($NaIO_4$) were acquired from Merck KgaA (Darmstadt, Germany).

2.2. Synthesis of DOPA-methyl ester

3,4-dihydroxy-L-phenylalanine (L-DOPA) was converted into L-DOPA methyl ester as described in the literature[1] . Briefly, L-DOPA (0.025 mol) in dry methanol (250 ml) was mixed with thionyl chloride ($SOCl_2$, 0.0505 mol) added in small quantities over 1 hr at 0 ^{0}C under Ar. After refluxing the mixture over 24 hours under Argon at 70 °C, toluene was added to the sample, and was rotavapped. Coevaporation with toluene was performed three times to remove the methanol (Figure S10). The ^{1}H-NMR (400 MHz, D_2O) spectra displayed the following peaks: δ = 4.64 (^{1}H,CH_2CHN), δ = 3.69 (3H, OCH_3), 3.04 (^{1}H, CH_aH_bCHN), 2.97 (^{1}H, CH_aH_bCHN) ppm.

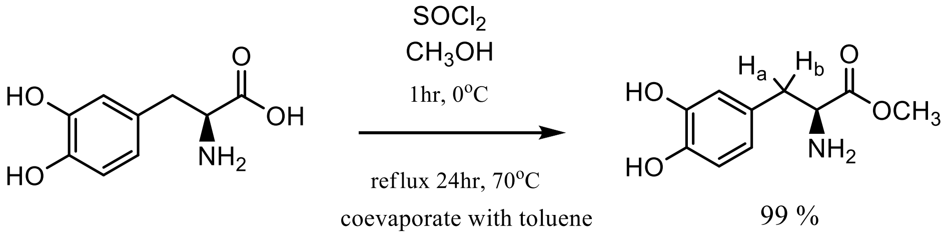

Figure S10: Reaction scheme for the preparation of DOPA-OMe.

2.3. Modification of Hyaluronic Acid with DOPA-OMe

Sodium hyaluronate (HA) (1 mg/ml , M_W : 132.3 kDa) was dissolved in MOPS buffer (0.1 M pH: 7.0) under Ar. 20-fold molar excess of N-hydroxysuccinamide (NHS) and 3,4-dihydroxy-L-phenylalanine-methyl ester (DOPA-OMe) were added to this solution. 1-Ethyl-3-[3-(dimethylamino)propyl]-carbodiimide (EDC) was dissolved in Milli-Q water, and added to the mixture (fresh EDC solution was added every 2 hours during the first 8 hours of the reaction to reach a final amount of 20-fold molar excess over the carboxylic acid groups of HA). The reaction was allowed to run overnight. The pH was checked to make sure it was below 7.0 since the catechol group of DOPA oxidizes at alkaline pH. The mixture was dialyzed (SnakeSkin, MWCO: 3.5 kDa) against Milli-Q water at pH: 3.5, and then freeze-dried. Modified HA was dissolved in 5% (w/v) NaCl solution and 10 volume equivalent ethanol was added to precipitate HA. After decanting

ethanol and redispersing the HA in water, the purified and precipitated polymer was again lyophilized. The reaction scheme is given in Figure S11. **Hyaluronic acid-catechol.** ^{1}H-NMR spectrum (400 MHz, D_2O): δ = 4.49 (^{1}H,CH_2CHN), 2.89 (^{1}H, CH_aH_bCHN), 2.86 (^{1}H, CH_aH_bCHN) ppm.

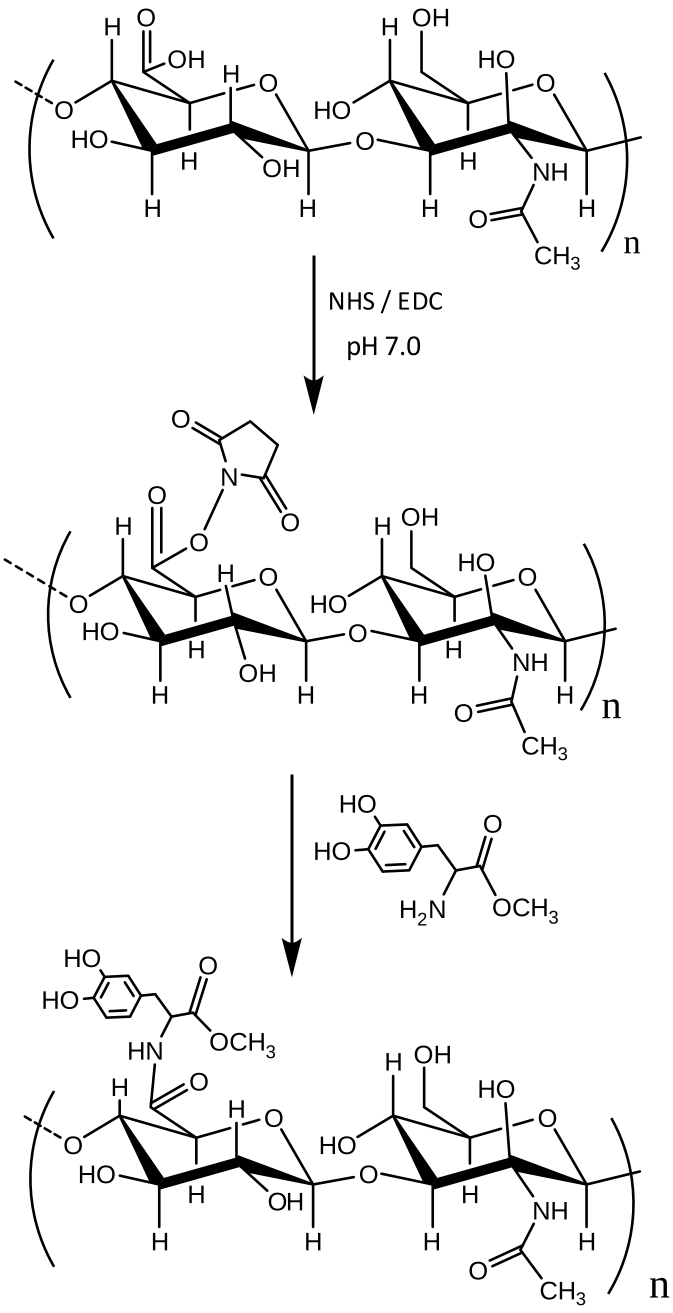

Figure S11: Reaction scheme for the modification of hyaluronic acid with DOPA methyl ester.

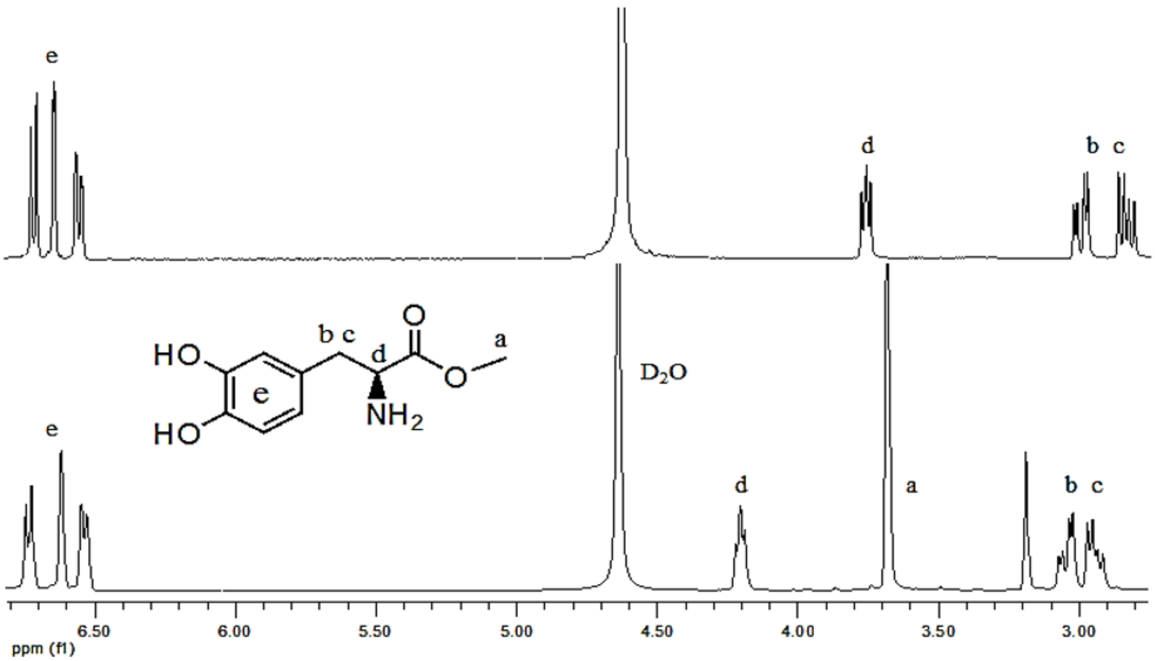

Figure S12: ^{1}H-NMR spectrum of L-DOPA (above) and L-DOPA Methyl Ester (below) in D_2O.

3. Characterization of Catechol-Modified HA and CHI

The degree of catechol modification was determined by UV-Vis spectrophotometry (Shimadzu UV-1700) using 3,4-dihydroxycinnamic acid (DOHA) as a calibration standard. DOHA (52.6 mg) was diluted to 25 ml with 0.015 M HCl solution and served as a stock solution. Then, 10x, 40x, 60x, 80x, 100x, 150x dilutions of stock solution were prepared. Absorbances at 280 nm and 320 nm (catechol and quinone absorb light at these wavelengths, respectively) were measured, and a calibration graph was drawn. The estimated degree of modification was 1.7 % (by mole) and 5.6% for CHI and HA, respectively.

4. Strain-Sweep Experiments

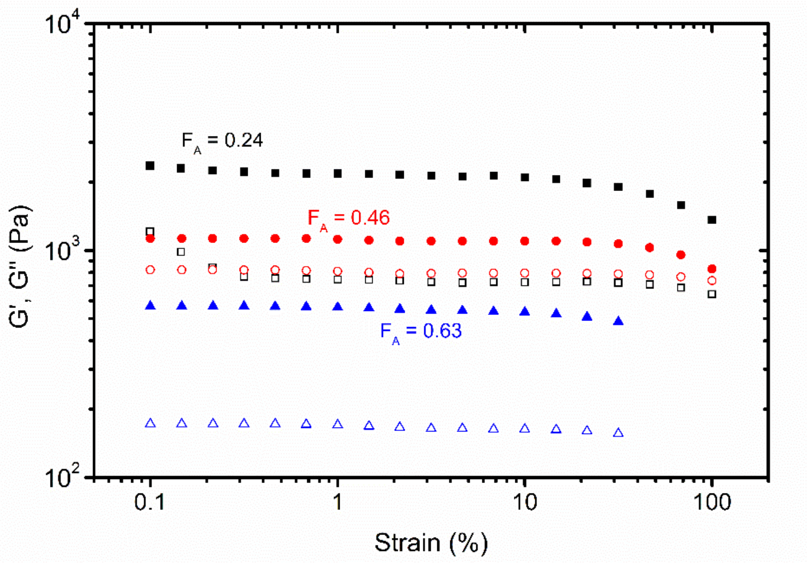

Figure S13: Strain sweep data, shown as the elastic modulus (G', closed data points) and viscous modulus (G'', open data points) vs. strain (%). Black squares, red circles, and blue triangles are data points for HA/CHI coacervates with F_A: 0.24, 0.46, and 0.63, respectively.

REFERENCES

71. Aubry, S.; Pellet-Rostaing, S; Lemaire, M. Oxidative Nucleophilic Substitution (SNOX) of the Benzylic Position as a Tunable Synthesis of Tetrahydroisoquinoline Natural Alkaloid Analogues. Eur. J. Org. Chem. **2007**, 2007(31), 5212-5225, doi: 10.1002/ejoc.200700366.

72. Liu, Z. Q.; Hu, B. H.; Messersmith, P. B. Convenient Synthesis of Acetonide Protected 3,4-Dihydroxyphenylalanine (DOPA) for Fmoc Solid-Phase Peptide Synthesis. Tetrahedron Lett. **2008**, 49(38), 5519-5521, doi: 10.1016/j.tetlet.2008.07.052.
